# Metagenome-Assembled Genome Sequences from an Anoxygenic Photosynthetic Consortium Involved in Sulfur Cycling

**DOI:** 10.1128/MRA.00819-20

**Published:** 2020-10-01

**Authors:** Lucia Winkler, Marc F. Münker, Susanne Brunotte, Lina Rohlmann, Alvaro Diez Alfageme, Anja Poehlein, Michael Hoppert, Joachim Reitner, Heiko Nacke

**Affiliations:** a FoLL Interdisciplinary Students’ Research Group, Faculty for Biology and Psychology and Faculty for Geosciences and Geography, Georg-August-Universität Göttingen, Göttingen, Germany; b Department of Genomic and Applied Microbiology, Institute of Microbiology and Genetics, Georg-August-Universität Göttingen, Göttingen, Germany; c Department of General Microbiology, Institute of Microbiology and Genetics, Georg-August-Universität Göttingen, Göttingen, Germany; d Geosciences Centre, Department of Geobiology, Georg-August-Universität Göttingen, Göttingen, Germany; University of Arizona

## Abstract

We sequenced the metagenome of an anoxygenic photosynthetic consortium originating from pond water and reconstructed four metagenome-assembled genomes. These genomes include *Desulfocapsa*, *Paludibacter*, *Lamprocystis*, and *Rhodocyclaceae* representatives and indicate the presence of genes for dissimilatory sulfate reduction and oxidation of reduced sulfur compounds.

## ANNOUNCEMENT

In the center of the former Willershausen clay pit in Germany (51°47′0.57ʺN, 10°6′50.85ʺE), ascending groundwater forms wetlands and ponds, which are hydrogen carbonate-sulfate/sulfate-calcium dominated because of a remolding in the underlying Zechstein salinar (1). Exclusively in the easternmost pond, globular aggregates of pink bacteria (“pink consortia”) were found in the water column.

In April 2019, a water sample was taken from the easternmost pond (oxygen content, 1.44 mg/liter; redox potential, −112.1 mV; a Xylem Analytics [Weilheim, Germany] FDO 925 oxygen sensor and an ORP 900 redox electrode were used for these measurements). To remove larger debris and arthropods, the sample was filtered through a stack of sieve pans with pore sizes of 500, 300, 80, and 41 μm (from coarse to fine). Fractions retained by the 80- and 41-μm meshes contained the pink consortia free from the aforementioned contaminants. DNA was extracted using the DNeasy PowerSoil kit according to the manufacturer’s protocol (Qiagen, Hilden, Germany). The purified DNA was used to generate Illumina paired-end sequencing libraries with the Nextera DNA sample preparation kit (Illumina, San Diego, CA, USA), and the libraries were sequenced by employing the MiSeq reagent kit v.3 and a MiSeq instrument, as stated by the manufacturer (Illumina). Default parameters were used for all software unless otherwise specified. Quality trimming of reads was performed by applying the program fastp v.0.19.4 (qualified quality Phred score, 20; minimum read length, 50 bp) (2) and yielded 26,465,778 paired-end reads. An average read length of 245 bp was recorded for the forward reads, and an average read length of 239 bp was recorded for the reverse reads. Base correction in overlapping regions (the correction option was selected with respect to fastp-based quality trimming using default parameters; this option allows identification of overlapping regions of each pair of reads, and mismatched base pairs in these regions can be corrected if one base shows high quality and the other very low quality) and removal of automatically detected adapter sequences were performed. Low-quality bases at the 5′ and 3′ ends of reads were trimmed once the mean quality within a sliding window of 4 dropped below 20. Sequences were *de novo* assembled into 5,933 contigs of ≥1,000 bp by utilizing metaSPAdes v.3.13.1 (3). The fastp and metaSPAdes outputs were fed into MaxBin v.2.2.7 for binning (minimum contig length, 1,000 bp; minimum probability for binning, 0.50) (4). Application of CheckM v.1.0.18 (5) revealed four relatively complete metagenome-assembled genomes (MAGs) (completeness, ≥94%; contamination rate, ≤2.1%). These four MAGs were annotated by using Prokka v.1.14.5 (6). Subsequently, the Prokka output was analyzed using the PathoLogic (7) component of the Pathway Tools software v.23.5 (8) with the MetaCyc database v.23.5 (9). Taxonomic analysis based on 16S rRNA gene sequences was performed using the Type (Strain) Genome Server (TYGS) v.205 (10). In addition, MAGs were classified taxonomically using GTDB-Tk v.1.0.2 and the Genome Taxonomy Database (GTDB) (release 89) (11, 12).

According to TYGS-based 16S rRNA gene analysis, MAG 01 (length of detected 16S rRNA gene sequence, 1,546 bp) is closely related to Desulfocapsa sulfexigens and MAG 03 (length of detected 16S rRNA gene sequence, 1,514 bp) to Paludibacter propionicigenes (with respect to MAG 02 and MAG 04, no 16S rRNA gene sequences were detected). GTDB-Tk-based classification confirmed that MAG 01 and MAG 03 are affiliated with *Desulfocapsaceae* and *Paludibacter*, respectively, and revealed that MAG 02 belongs to *Lamprocystis* and MAG 04 belongs to *Rhodocyclaceae*. Functional analysis revealed the presence of genes for dissimilatory sulfate reduction, encoding, e.g., dissimilatory adenylyl-sulfate reductases (AprA and AprB) and sulfite reductases (DsvA, DsvB, DsvC, and AsrA), in MAGs 01, 02, and 04. All four MAGs encode the polysulfide reductase NrfD, indicating potential polysulfide formation from sulfur. MAG 02 and MAG 04 harbor genes for oxidation of reduced sulfur compounds, e.g., genes encoding the sulfide dehydrogenase subunits FccA and FccB and the sulfite dehydrogenase SoeB. Furthermore, both MAGs contain genes encoding parts of a Sox system (*soxA* and *soxY*).

### Data availability.

Raw sequencing data are available at the NCBI Sequence Read Archive (SRA) under accession number SRR11932628. The metagenome assembly is available at GenBank under accession number JACDZJ000000000. The MAGs are available at GenBank under accession numbers JACDZF000000000, JACDZG000000000, JACDZH000000000, and JACDZI000000000. Prokka-based annotations of contigs with respect to the four MAGs are publicly available at the Göttingen Research Online Database (https://doi.org/10.25625/EZNVW8).
